# Organotypic culture system for prepubertal mice testicular tissue: A comparative study

**DOI:** 10.3389/fendo.2025.1664628

**Published:** 2025-09-25

**Authors:** Liping Deng, Xinyi Wang, Xunwei Wu, Hui Zhou, Yintao Cheng, Honggang Li

**Affiliations:** ^1^ Institute of Reproductive Health, Tongji Medical College, Huazhong University of Science and Technology, Wuhan, China; ^2^ Key Laboratory for Major Obstetric Diseases of Guangdong Province, The Third Affiliated Hospital of Guangzhou Medical University, Guangzhou, China; ^3^ Department of Urology, Wuhan Children’s Hospital (Wuhan Maternal and Child Healthcare Hospital), Tongji Medical College, Huazhong University of Science and Technology, Wuhan, China

**Keywords:** prepubertal boy, testicular organotypic culture, culture systems, germ cells, Sertoli cells

## Abstract

**Background:**

Prepubertal testicular organotypic culture serves as an *in vitro* model for research in regenerative medicine, developmental biology, and toxicology. However, the selection of culture systems for diverse applications and purposes lacks explicit rationale or standardized guidelines.

**Objective:**

To evaluate the developmental status of germ cells and Sertoli cells in prepubertal testicular organotypic cultures using different media and tools, and to provide evidence for model standardization.

**Materials and Methods:**

Immature testicular tissues (ITTs) were isolated from prepubertal mice and cultured in combinations of three media (Medium 1/2/3, M1/2/3) and two tools (agarose gel pillars, tissue culture inserts) at 34°C with 5% CO_₂_ for 8 days. After culture, tissue morphology and cell status were assessed via histological analysis (hematoxylin-eosin [HE] staining) and immunofluorescence. Quantification was performed using ImageJ, and statistical analysis was conducted with GraphPad Prism.

**Results:**

Over the 8-day culture period, ITTs survived and grew in all systems, maintaining the structural integrity of seminiferous tubules, with tubular diameter increasing over time. Spermatogonia remained viable within the tubules, the number of germ cells increased, and both germ cells and spermatogonia exhibited proliferative activity. Sertoli cells were well preserved, and spermatogonia differentiated into meiotic spermatocytes in the tubule lumen. Compared with M1, M2 and M3 significantly enhanced the proliferation rates of germ cells and spermatogonia, with M2 showing the highest efficiency. Notably, M2 yielded the greatest number of differentiated spermatocytes, whereas M1 better preserved spermatogonial populations despite its lower proliferation rate.

**Conclusion:**

This study clarifies the differential effects of culture systems on the development of germ cells and Sertoli cells in ITTs, and provides a standardized organotypic platform for investigating spermatogenesis, male infertility, and potential therapeutic interventions.

## Introduction

The organotypic culture model is a rapidly developing field in *in vitro* biological science. The cultivation techniques have evolved from initial explorations to multidisciplinary integration, gradually becoming a powerful tool for simulating *in vivo* environment (1, 2). Compared to traditional two-dimensional (2D) cell culture methods, three-dimensional (3D) organotypic culture models can promote cell differentiation and more accurately reproduce the 3D structures and organ-specific functions found *in vivo* (3). In recent years, organotypic cultures have been widely applied to various tissues, including the testis, airway, cornea, intestine, kidney, neurons, oral tissues, skin, and tumors (4).

The testis is a vital organ of the male reproductive system, responsible for sperm production and androgen secretion (5, 6). Organotypic culture of testicular tissue is currently considered the most physiologically relevant *in vitro* model of the testis, as it effectively replicates the complex structure of seminiferous tubules (STs) while preserving the testicular cellular niche. Although this technique is not yet fully optimized, it has made significant progress and demonstrated success in various applications, including reproductive developmental biology, toxicology, fertility preservation, and regenerative medicine (7, 8). This includes replicating the 3D structure and microenvironment of the testis to investigate cellular interactions and signaling mechanisms during testicular development; establishing *in vitro* models to gain deeper insights into disease pathogenesis and identify potential therapeutic targets (9); assessing the toxicological effects of chemicals, drugs, or environmental factors on testicular tissue to evaluate their potential risks to male reproductive health; and leveraging testicular organoids in drug development to screen for compounds affecting the reproductive system, enabling the evaluation of their efficacy and safety (10–12).

In recent years, organotypic culture of testicular tissue has emerged as a promising strategy for fertility preservation in prepubertal males. Prepubertal boys subjected to gonadotoxic therapies (*e.g.*, chemotherapy/radiation) or those with reproductive disorders (*e.g.*, Klinefelter syndrome) are at high risk of infertility due to germ cell loss. However, their immature testes generally lack spermatogenic capacity, precluding sperm cryopreservation as a viable preservation approach (13). As a result, fertility preservation is essential for these individuals. Prior to treatment, testicular tissue containing spermatogonial stem cells (SSCs) is cryopreserved for potential future fertility restoration. There are three main strategies for restoring fertility: SSCs transplantation, testicular tissue transplantation, and *in vitro* SSCs/ITTs culture. Among these approaches, organotypic culture of ITTs stands out due to its significant advantages and is frequently employed in preclinical fertility restoration research. It serves as a valuable tool for exploring the potential of *in vitro* spermatogenesis in mice, offering innovative strategies to address male infertility, particularly in prepubertal males (14, 15).

Over the past decade, *in vitro* culture techniques have progressively improved and optimized, including the development of physical support systems such as hanging drop cultures, metal mesh grids, agarose blocks, mesh membranes, and synthetic scaffolds. Additionally, rich and complex culture media have been developed to support growth and development, incorporating components such as growth factors, hormones, small molecules, antioxidants, and lysophospholipids (16, 17). Optimal environmental conditions for culture, including temperature, humidity, oxygen and carbon dioxide levels, and pH, have also been explored. Moreover, organotypic cultures of testicular tissue have been increasingly employed in tissue engineering, simulating *in vivo* conditions with advanced technologies such as microfluidic devices (11, 18).

Although current techniques have indeed achieved considerable accomplishments and progress, further refinement and improvement is required to ensure the model’s applicability. For testicular tissue culture, various studies have employed different culture systems. For instance, de Michele et al. employed DMEM/F-12 medium supplemented with 10% (v/v) KnockOut™ Serum Replacement (KSR) as the basal culture system, utilizing Millicell^®^ cell culture inserts to establish an organotypic culture model for prepubertal testicular tissues. This experimental design enabled evaluation of the differentiation potential of human ITTs (19). Similarly, Delessard and colleagues used α-MEM supplemented with 10% (v/v) KSR and Agarose Gel Pillars (AGPs) for the *in vitro* culture of ITTs to evaluate the impact of chemotherapy on *in vitro* spermatogenesis (20, 21). These studies did not make clear distinctions between the culture systems, particularly regarding how to select appropriate systems tailored to specific purposes. Therefore, designing matched culture systems for different purposes is crucial to further refining the organotypic culture model of testicular tissue, making it more precise and effective.

In this study, we summarized the commonly used culture media and tools in testicular organotypic culture research, and selected three representative media and two typical tools. Using a standard air-liquid interface culture method with fresh testicular tissue fragments from postpartum day 5 days postpartum (5dpp) mice, we performed short-term cultures to monitor the growth and development of testicular cells, particularly spermatogonia. The objective was to analyze and compare the differential effects of the three culture systems on the growth and development of testicular tissues, thereby facilitating the determination of their distinct applications.

## Materials and methods

### Collection of mouse testicular tissue

The animal studies were approved by the Institutional Animal Care and Use Committee (IACUC) of HUAZHONG University of Science and Technology, and all procedures were performed in accordance with the Regulations of Experimental Animal Administration issued by the State Committee of Science and Technology of the People’s Republic of China. C57BL/6J mice were acquired from Hubei Biont Biological Technology Co., Ltd (SCXK(e)2021-0027, SYXK(e)2021-0019), and housed at a temperature of 22-23°C with appropriate humidity under a 12-hour light/dark cycle. Male mice at 5dpp were euthanized by decapitation. Both testes were surgically removed, and immediately placed in an ice-cold phosphate-buffered saline (PBS) solution containing 1% penicillin (100IU/mL) streptomycin (0.1mg/mL) Under a dissecting microscope, the tunica albuginea was carefully removed using fine forceps and scissors. Subsequently, the testicular parenchyma was dissected into fragments to initiate the culture of fresh testicular tissue.

### Preparation of agarose gel pillars

AGPs (1.5% concentration) was prepared as follows: Agarose (1.5g) was dissolved in 100 ml of distilled water (22, 23). After autoclaving at high pressure for 30 minutes, 33 ml of the agarose solution was poured into a 10 cm-diameter culture dish for cooling. The agarose solution was allowed to cool until it forms a 5mm thick gel layer (16). Using a sterile surgical knife with the assistance of a grid ruler, the agarose gel was cut into 1cm^2^ cubic shapes. These cubic gel blocks were then placed into a 24-well plate (3524, CORNING), ensuring they were fully covered with culture medium, and incubated overnight in a cell culture incubator.

After 24 hours, the medium in the 24-well plate was replaced, ensuring that the gel pillars were not fully submerged in the medium. ITT fragments (1–3 pieces) were put on each AGPs, ensuring that the ITT tissue fragments did not directly contact the culture medium.

### Organotypic cultures at a gas-liquid interphase

Following a previously established protocol (24), organotypic tissue cultures were conducted *in vitro*. Briefly, testicular tissue from male mice at 5dpp was dissected into 1–3 mm fragments in diameter and around 200 µm in thickness. These fragments were either placed on 1.5% AGPs (1110GR100 BioFroxx) within 24-well plates, with the AGPs submerged halfway in culture medium, or single tissue fragments placed on 24-well Tissue Culture Inserts (14312, LABSELECT). In the latter setup, tissues were placed on polyester membranes in the upper compartment, while the lower compartment contained 300 µl of culture medium, which was replaced every 3–4 days. This volume ensures the medium only contacts the basal surface of the testicular tissue, maintaining proper air-liquid interface culture conditions. The culture condition was a humidified incubator with 5% CO_2_ at 34°C.

### Composition of different culture systems

**Figure 1:**
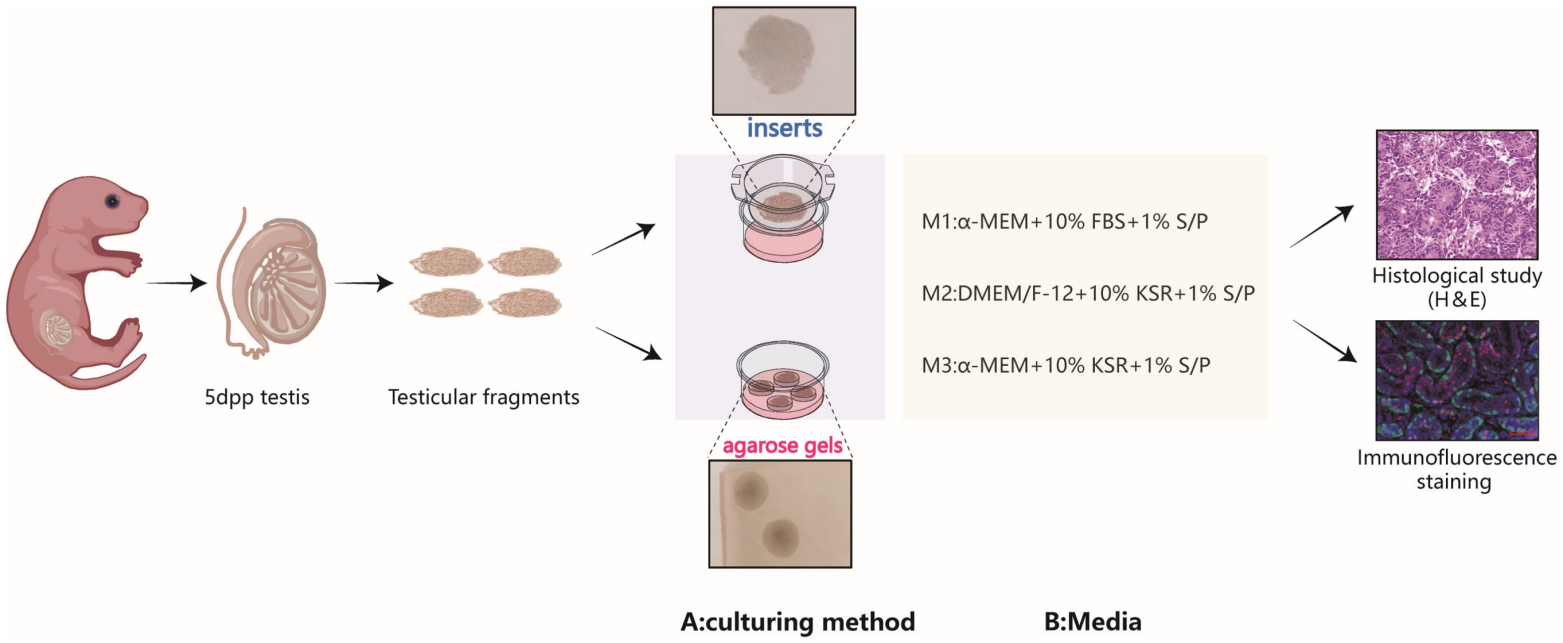
Schematic representation of the experimental design. The immature testicular tissues, sourced from testes of 5dpp pups, were fragmented into testicular tissue pieces and reassembled under various culture tools and medium compositions, hematoxylin-eosin staining and immunofluorescence are used to evaluate cultured tissues.

Three different culture media were used, oesignated as M1, M2, and M3 (**Figure 1**). M1: Comprised of MEMα, GlutaMAX™ additive, nucleoside-free (32561037, Gibco), supplemented with 10% fetal bovine serum (FBS) (Every Green, 70220-8611), and 1% penicillin/streptomycin ( S/P). M2: Consisted of Dulbecco’s Modified Eagle Medium: Nutrient Mixture F-12 (DMEM/F-12), KSR (10828028, Gibco), and 10% S/P. M3: Included MEMα supplemented with KSR and 10% S/P.

### Histological analysis

During the culture period, testicular tissue was collected bi-daily and fixed in Bouin’s solution or 4% paraformaldehyde. Each specimen was sectioned to display slices with the maximum surface area, with a section thickness of 5 µm, and stained with hematoxylin-eosin. For each fragment or testis in each group, 30 cross-sections of ST were analyzed in each section, with a minimum interval of 35 µm between sections, to obtain an overall assessment of the tissue. We used the Pannoramic SCAN slide scanner (3D Histech, Hungary) to analyze the evolution of the average diameter of intact ST cross-sections. The maximum diameter of the STs (measured from one side of the basement membrane to the other) was recorded, and changes in ST diameter were analyzed across different culture systems and over time.

### Immunofluorescence staining analysis

Tissue sections were deparaffinized, hydrated, and rinsed in PBS for 3 minutes. Sections were subjected to high-pressure antigen retrieval in pH 6.0, 0.01M citrate buffer (G1202, Servicebio) at 125°C for 30 minutes. After cooling to room temperature, sections were washed three times for 5 minutes each in distilled water. Sections were permeabilized with 0.1% (v/v) Triton X-100 at room temperature for 15 minutes, followed by blocking with 5% (w/v) bovine serum albumin (Sigma-Aldrich). Slides were then incubated with primary antibodies overnight at 4°C in a humidified chamber. After 16 hours, sections were washed three times with PBS containing 0.05% Tween-20 and then incubated with appropriate secondary antibodies. Negative controls were incubated with isotype IgG of the same species as the primary antibody. After incubation, sections were washed and counterstained with DAPI for 6 minutes to stain nuclei. Sections were then washed, air-dried, and mounted with an anti-fade mounting medium.

Immunofluorescence was used to identify undifferentiated spermatogonia (anti-PLZF, anti-SALL4), germ cells (anti-DDX4, also known as VASA), Sertoli cells (anti-GATA4), meiotic germ cells (anti-SYCP3), and proliferating germ cells using double immunofluorescence (anti-KI67, anti-PCNA). Additionally, the identification and localization of meiotic (anti-DDX4^+^ and anti-SYCP3^+^) germ cells were confirmed. All analyses were performed on randomly selected sections, with a minimum interval of 50 µm between sections. Images were captured at 400× magnification using a Carl Zeiss Apotome.3 microscope (Axio Observer 5). Cell counting was performed using Image J software by quantifying immunofluorescence signals from secondary antibodies with distinct color channels. To assess proliferation efficiency, we calculated the ratio of double-positive to single-positive cells. Specifically, for Ki67 and DDX4 analysis, we determined the percentage of Ki67+/DDX4+ double-positive cells relative to all Ki67+ cells within seminiferous tubule lumens based on positive fluorescence signals. Additionally, we quantified relative cell numbers by measuring positive fluorescence signals per unit area, as employed for PLZF+ cell enumeration. The density of tubular markers (PLZF, DDX4, GATA4) was calculated by determining the total number of each cell type relative to the total area of ST cross-section (mm²), with manual cell counting for comparison. For the analysis of proliferating germ cells, KI67^+^ (proliferating cells) and double PLZF^+^KI67^+^ (proliferating SSCs) cells were manually counted. For the analysis of meiotic germ cells, DDX4^+^ (germ cells) and double DDX4^+^SYCP3^+^ (spermatocytes) cells were manually counted.

For IF controls, neonatal mouse (SALL4, PLZF, KI67, PCNA) and mature testicular tissue (SYCP3, DDX4, GATA4) were used as positive controls, and isotype IgG antibodies were used as negative controls (**Supplementary Figure 1**). Details of the primary antibodies used in the experiments can be found in **Supplementary Table 1**.

The analysis was performed as follows: Per biological replicate (n=6 per group): Each slide was examined, and 3–5 random high-power fields (HPFs, 40×) were selected. Within each HPF, all intact and visible seminiferous tubules (STs) were counted, and 10 STs per HPF were systematically evaluated (e.g., for diameter, germ cell layers, etc.). Total STs per group: This design resulted in 30–50 STs evaluated per biological replicate (3–5 HPFs × 10 STs). Thus, each experimental group (n=6) included 180–300 STs in total (30–50 STs × 6 replicates).

### Data analysis

All experiments included at least 6 biological replicates (n=6), where each replicate represented a tissue sample obtained from an independent animal. To control for inter-individual variability, each experimental group included tissue samples from six distinct individuals. Data were analyzed using the GraphPad Prism 8 software. Statistical analyses were performed using GraphPad Prism 8, and data are presented as the mean ± standard deviation (Mean ± SD) for normally distributed data. The normality of data distribution was assessed using the Shapiro-Wilk test. For multiple group comparisons, one-way or two-way analysis of variance (ANOVA) was applied, followed by Bonferroni correction for pairwise comparisons. To evaluate the effects of culture time and system on the dependent variables, a two-way ANOVA model was used. Statistical significance was defined as p < 0.05.

## Results

### ITT histological evaluation

**Figure 2:**
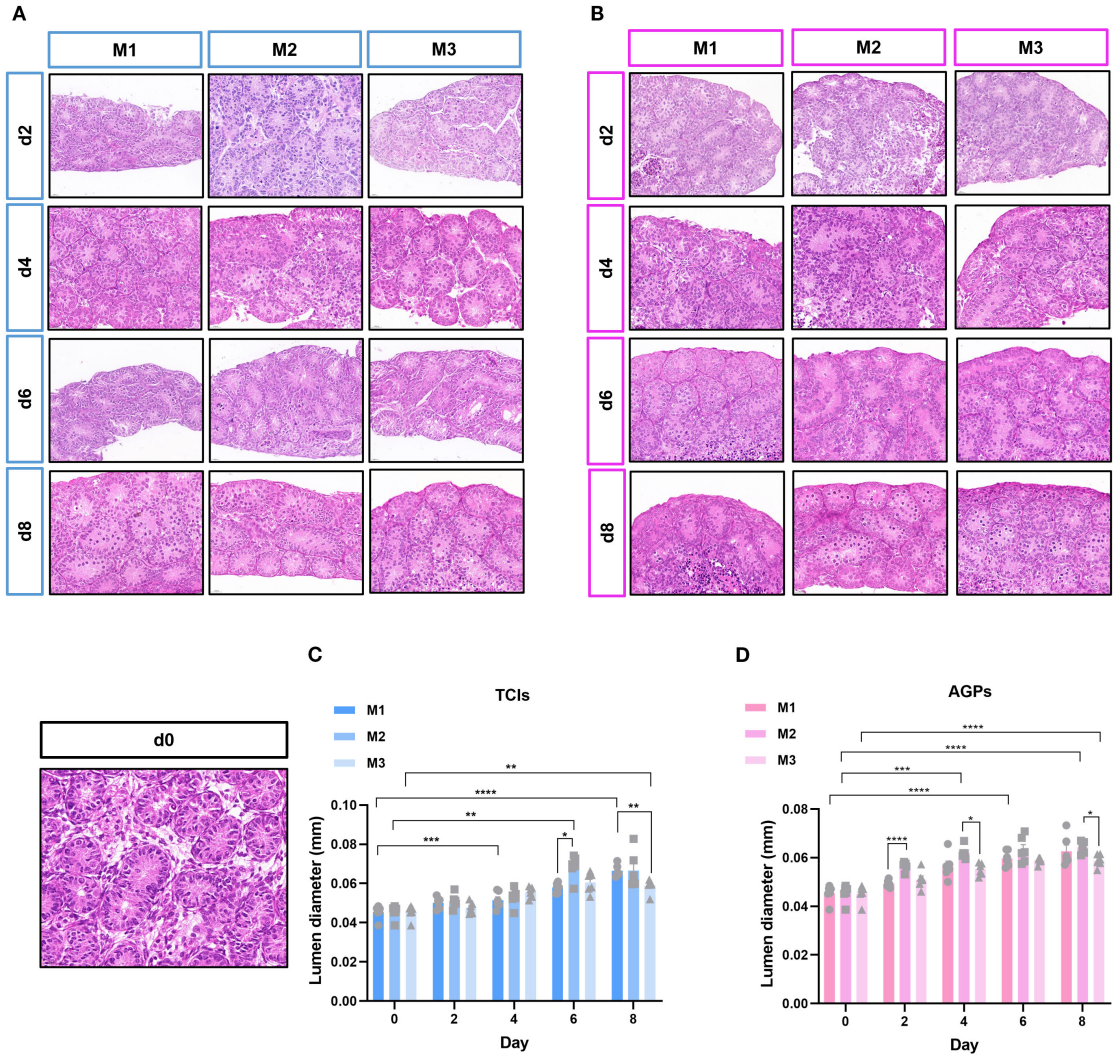
The evolution of ST cross-sectional diameter during cultivation. Tissue fragments were cultured in Tissue Culture Plant Insert **(A)**, and in AGPs **(B)**, Histological view of the cultured testis tissue. **(C, D)** Evolution of mean ST cross-section diameter in the different systems over the culture period, the transverse diameter (circular cross-section) of seminiferous tubules (STs) was selected for statistical analysis. Each data point represents an independent biological replicate (n=6), derived from tissue samples of six animals, data are presented as mean ± standard deviation. Statistical significance between groups was assessed using ANOVA, Statistically significant differences are indicated (*p ≤ 0.05, **p ≤ 0.01, ***p≤0.001, ****p ≤ 0.0001). Scale bars = 20μm.

During ITT culture, the tissue morphology and the structure of ST remained intact. The changes in the diameter of STs across different culture systems during the culture period are shown in **Figures 2A, B**. As expected, the culture duration significantly influenced the tubular diameter. Regardless of the culture tool used, the luminal diameter of the ST increased from day 4 to day 8 in all systems. Interestingly, in the M2 culture system, the enlargement of ST lumen was observed earlier. On day 2, the tubular diameter in M2 cultured with AGPs was significantly larger than that in M1 (p < 0.0001, **Figures 2C, D**). In conclusion, these data indicate that all culture systems are capable of maintaining ITT morphology and supporting the enlargement of ST lumens.

### The amount and proliferation of germ cells

**Figure 3:**
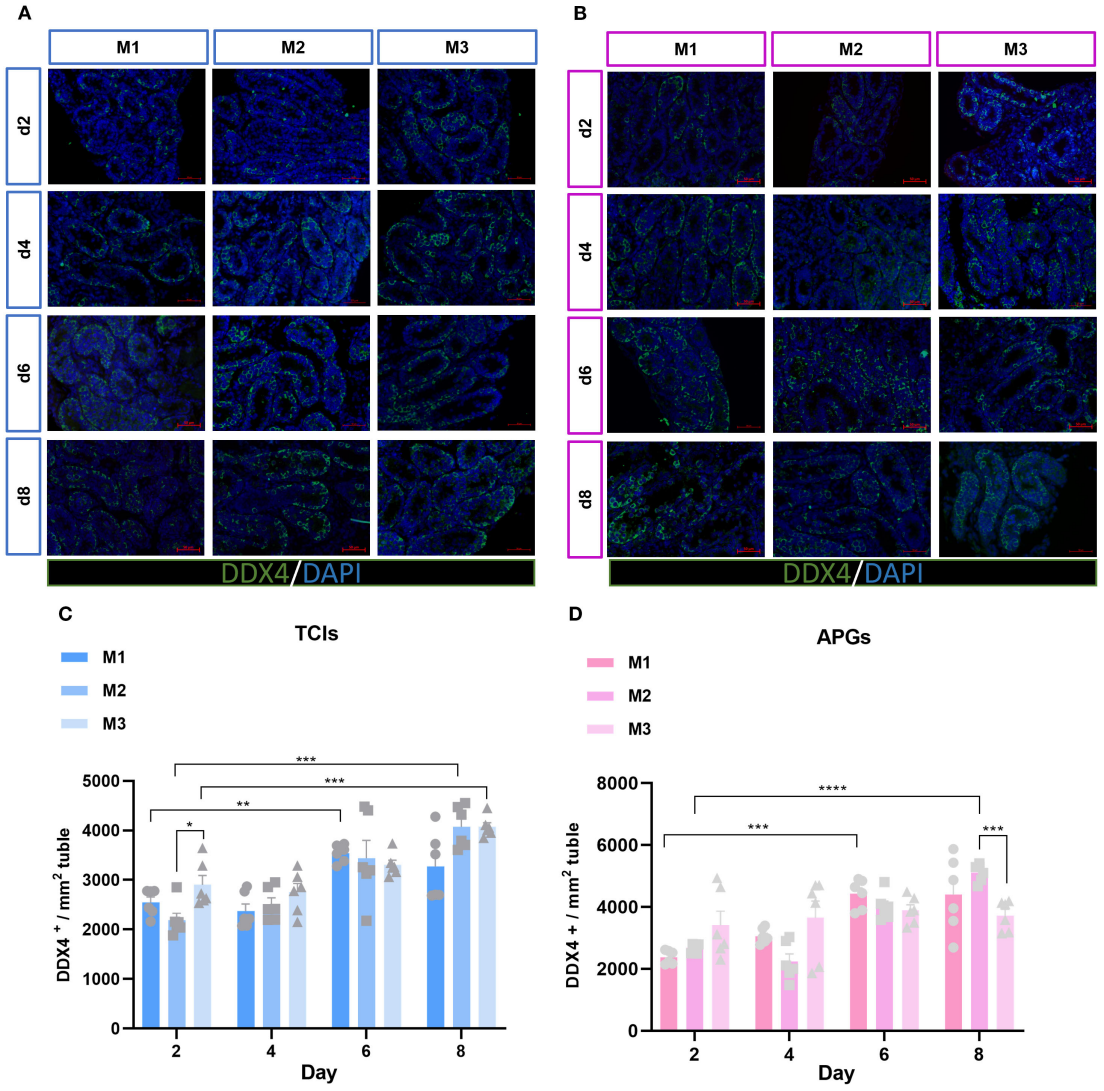
Evolution of germ-cell numbers mm² of ST over the culture period. **(A, B)** Tissue fragments were cultured in Tissue Culture Plant Insert **(A)**, and in AGPs **(B)**, immunofluorescence staining of DDX4 Positive Cells. **(C, D)** Evolution of the number of DDX4 positive cells mm² of ST in prepubertal mice on day 2,4,6 and 8 over the culture period. Images shown at ×400 magnification. Scale bars = 50μm. Each data point represents an independent biological replicate (n = 6) from six animals. Data are presented as mean ± standard deviation. Statistical significance was assessed using ANOVA (*p ≤ 0.05, **p ≤ 0.01, ****p ≤ 0.0001).

**Figure 4:**
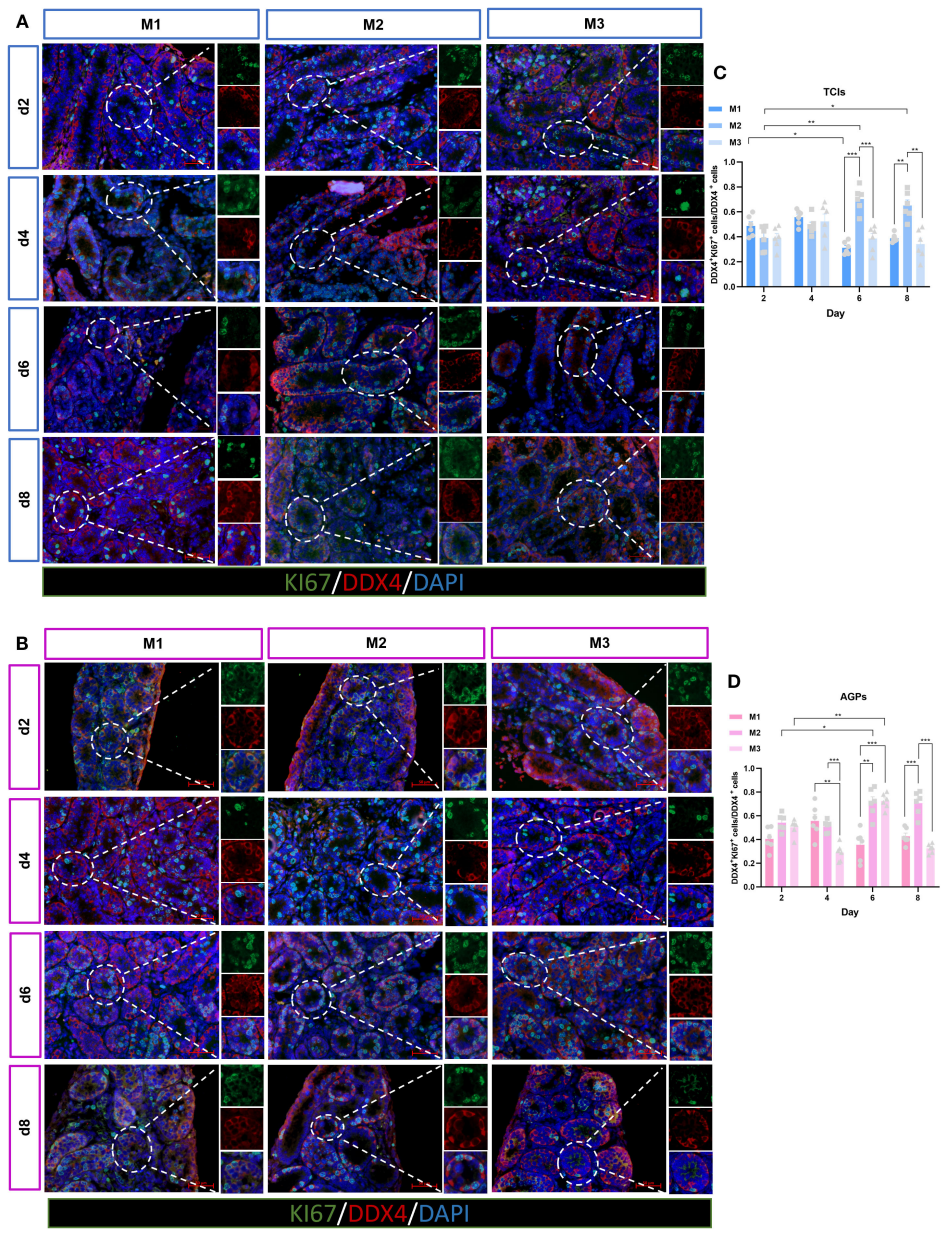
Evolution of germ cell proliferation over the culture period. **(A, B)** Tissue fragments were cultured in Tissue Culture Plant Insert **(A)**, and in AGPs **(B)**, immunofluorescence duplex staining for DDX4/Ki67. **(C, D)** Evolution of the number of proliferating Germ cells (DDX4^+^Ki67^+^ cells/Ki67^+^) over the culture period. Images shown at ×400 magnification. Scale bars = 50μm. Data are presented as mean ± SD. Statistical significance was determined using ANOVA (*p ≤ 0.05, **p ≤ 0.01).

To investigate the status of germ cells in ST under different culture systems and time points, we analyzed the germ cell density (cells/mm²) in each ST. Significant differences were observed in the average number of germ cells (DDX4^+^) mm² of ST cross-section across different time points and culture systems (**Figures 3A, B**). With prolonged culture duration, the number of germ cells gradually increased. Notably, DDX4^+^ germ cells showed statistically significant differences on day 6 and day 8 among the different culture tools (**Figures 3C, D**). The proliferation of germ cells within the tubular lumen was further determined (**Figures 4A, B**). As expected, the results revealed that different culture systems had a significant impact on germ cell proliferation (DDX4^+^+KI67^+^/DDX4^+^). In tissues cultured using Tissue Culture Inserts (TCIs), the proliferation rate of DDX4^+^ cells in the M2 system were significantly higher than that in the M1 and M3 on day 6 and day 8. Additionally, in tissues cultured with AGPs, the M2 exhibited a higher proliferation rate of DDX4^+^ cells on days 4, 6, and 8 (**Figures 4C, D**). These results indicated that germ cells can maintain a certain level of viability and functionality *in vitro* throughout the culture period. Furthermore, the proliferation rate of germ cells increased over time in the M2 and was consistently higher when compared to the M1 and M3.

### Sertoli cell number

**Figure 5:**
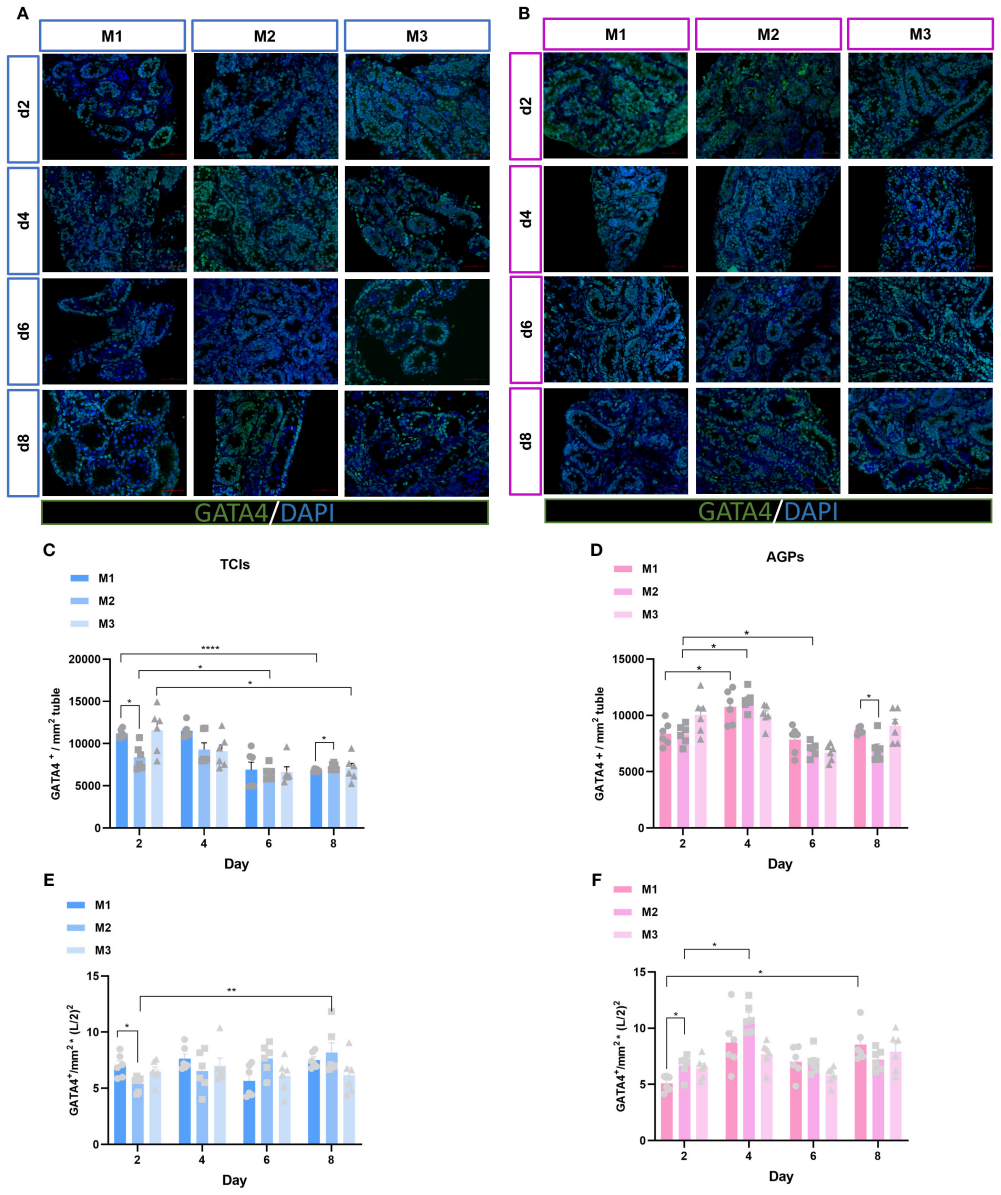
Evolution of Sertoli cell numbers mm²of ST over the culture period. **(A, B)** Tissue fragments were cultured in Tissue Culture Plant Insert **(A)**, and in AGPs **(B)**, immunofluorescence staining of GATA4 Positive Cells. **(C, D)** Evolution of the number of GATA4 positive cells mm²of ST over the culture period, **(E, F)** The number of Sertoli cells was quantitatively analyzed. L represents lumen diameter, measured in millimeters (mm). Images shown at ×400 magnification. Scale bars = 50μm. Data are presented as mean ± SD. Statistical significance was determined using ANOVA (*p ≤ 0.05, **p ≤ 0.01, ****p ≤ 0.0001).

Sertoli cells represent the sole type of somatic cells within the spermatogenic epithelium, playing a critical role in supporting and regulating spermatogenesis. To further elucidate the status of Sertoli cells in this context, their quantity was assessed using the GATA4 marker at various time intervals and across different culture systems (**Figures 5A, B**). The average number of Sertoli cells mm² of ST cross-section was analyzed. Surprisingly, both culture duration and system significantly influenced the number of Sertoli cells. During the entire culture period, regardless of the tools used, the number of GATA4^+^ cells in all three systems showed a declining trend as culture time increased, particularly on days 6 and 8, when the average number of GATA4^+^ cells in the M2 culture system exhibited a significant decrease (**Figures 5C, D**). To further determine whether there was a loss of Sertoli cells during *in vitro* culture, we calculated Sertoli cell quantity by multiplying the square of the tubular radius from **Figure 2** with the number of GATA4^+^ Sertoli cells mm² of ST cross-section. The data demonstrated that Sertoli cell numbers were maintained throughout the culture period. Moreover, in the M1 and M2 systems, Sertoli cell numbers increased over time (**Figures 5E, F**). To validate these findings, we conducted immunofluorescence staining on testicular tissues from genetically identical mice at postnatal days 3, 5, 8, and 10. The results consistently demonstrated reduced ratios of GATA4^+^ cells/mm² tubule area and SOX9^+^ cells/mm² tubule area (**Supplementary Figure 2**). These data collectively confirm that the Sertoli cell population in our *in vitro* cultured testicular tissues was not reduced but remained well-preserved. These results indicate that Sertoli cells were not lost during *in vitro* culture but rather increased in number as the culture duration extended.

### Quantity and proliferation of spermatogonia

**Figure 6:**
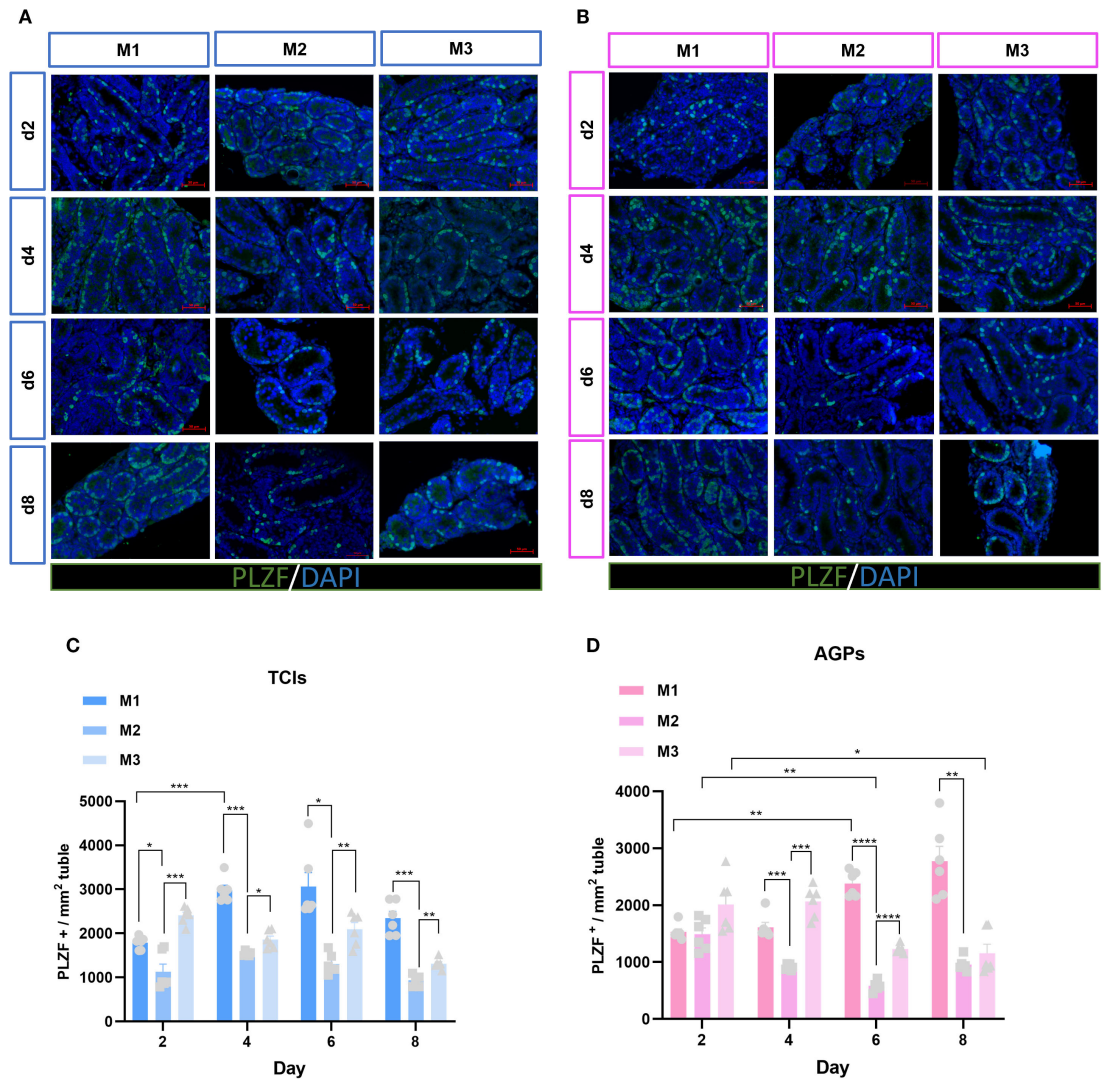
Evolution of spermatogonia numbers mm²of ST over the culture period. **(A, B)** Tissue fragments were cultured in Tissue Culture Plant Insert **(A)**, and in AGPs **(B)**, immunofluorescence staining of PLZF Positive Cells. **(C, D)** Evolution of the number of PLZF positive cells mm²of ST over the culture period. Images are shown at ×400 magnification. Scale bars = 50μm.Data are presented as mean ± SD. Statistical significance was determined using ANOVA (*p ≤ 0.05, **p ≤ 0.01, ****p ≤ 0.0001).

Spermatogenesis is a complex process that occurs within the STs, with two key stages being the maintenance and proliferation of spermatogonia, and the differentiation of spermatogonia into spermatocytes. To further investigate the spermatogonia within the tubules, we analyzed the average number of spermatogonia (PLZF^+^) mm² of ST cross-section (**Figures 6A, B**). PLZF is predominantly expressed in undifferentiated spermatogonial stem cells (25). Surprisingly, in the M1 system, the number of spermatogonia significantly increased from day 4 to day 8, exceeding that in the M2 and M3, especially surpassing the quantity observed in the M2. In contrast, spermatogonia numbers in the M3 and M2 slightly decreased over time (**Figures 6C, D**).

**Figure 7:**
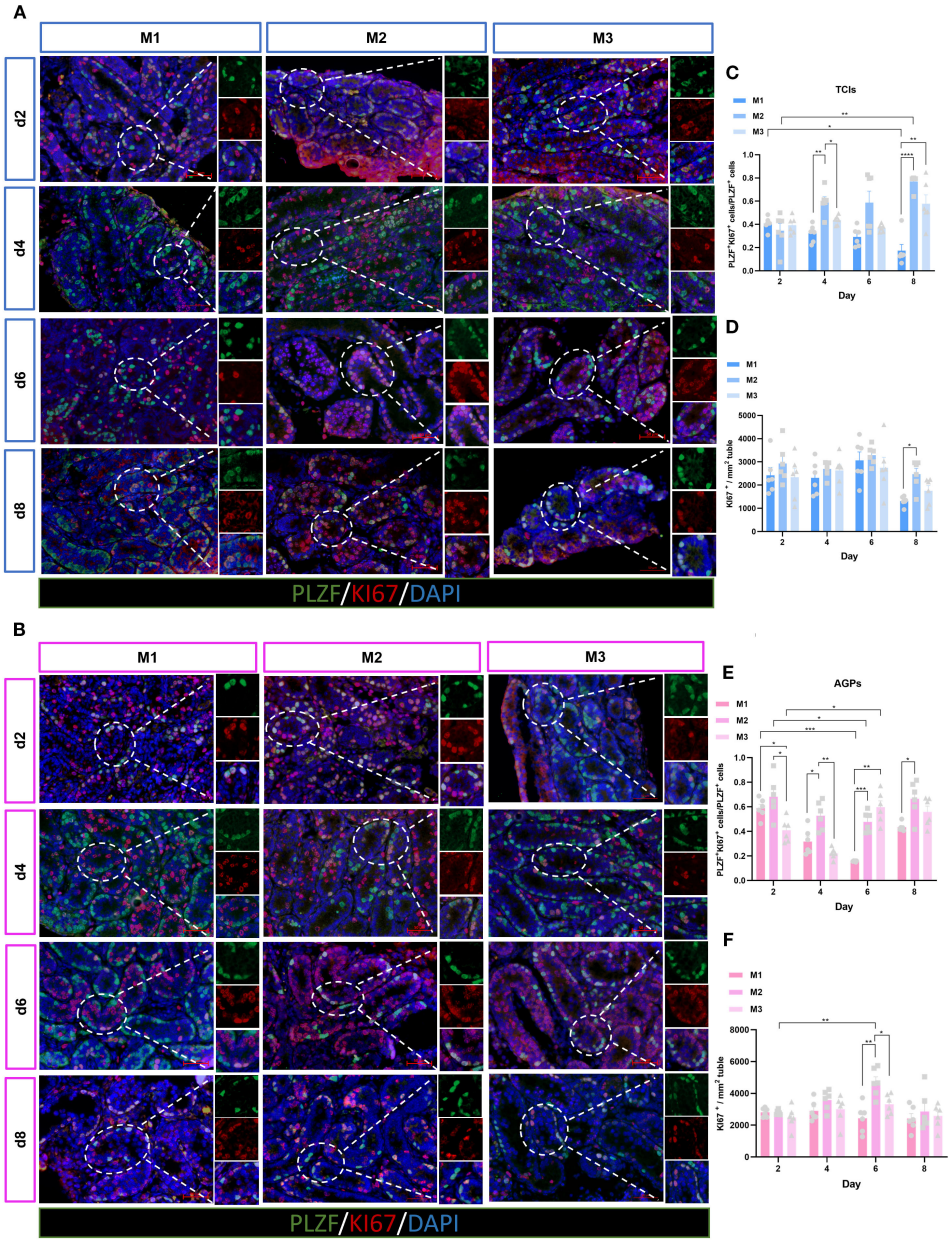
Evolution of spermatogonia cell proliferation over the culture period. **(A, B)** Tissue fragments were cultured in Tissue Culture Plant Insert **(A)**, and in AGPs **(B)**, immunofluorescence duplex staining for PLZF/Ki67. **(C, E)** Evolution of the number of proliferating Spermatogonia cells (PLZF^+^+Ki67^+^/PLZF^+^) over the culture period. **(D, F)** Evolution of the number of KI67-positive cells mm² in ST. Images are shown at ×400 magnification. Scale bars = 50μm. Statistical significance was determined using ANOVA (*p ≤ 0.05, **p ≤ 0.01, ****p ≤ 0.0001).

The maintenance and proliferative renewal of spermatogonia are critical for spermatogenesis. We evaluated the proliferation of spermatogonia across different culture systems and time points. Unexpectedly, the proliferation of spermatogonia was significantly influenced by both the culture system and time (**Figures 7A, B**). Regardless of the culture tool used, the proliferation rate of spermatogonia (PLZF^+^+KI67^+^/PLZF^+^) in the M1 system decreased gradually over time. In M1, when using AGPs, the proliferation rate on day 6 was significantly lower than on day 2 (p < 0.05). Among the three culture systems, the proliferation rates of spermatogonia in M2 and M3 were consistently higher than in M1, with M2 exhibiting the highest proliferation rate, which appears contrary to the observed spermatogonia quantity (**Figures 7C, E**). Additionally, we analyzed all proliferative cells within the tubules. The data showed that with prolonged culture, M2 maintained a higher number of proliferative cells (**Figure 7D, F**). SALL4 is a marker of spermatogonial pluripotency and exhibits elevated expression levels in spermatogonial stem cells as well as during the initial stages of their differentiation (26). The SALL4^+^+PCNA^+^/SALL4^+^ results indicated that spermatogonia could maintain a certain level of proliferation during *in vitro* culture (**Supplementary Figure 3**). Our data suggest that during *in vitro* culture, the number of spermatogonia in M2 and M3 decreased over time, with the most pronounced reduction observed in M2. However, spermatogonia in the M2 exhibited more robust proliferation, maintaining an active proliferative state throughout the culture period. In contrast, the M1 system shows better maintenance of spermatogonial numbers, albeit with lower proliferation rates.

### Differentiation of germ cells

**Figure 8:**
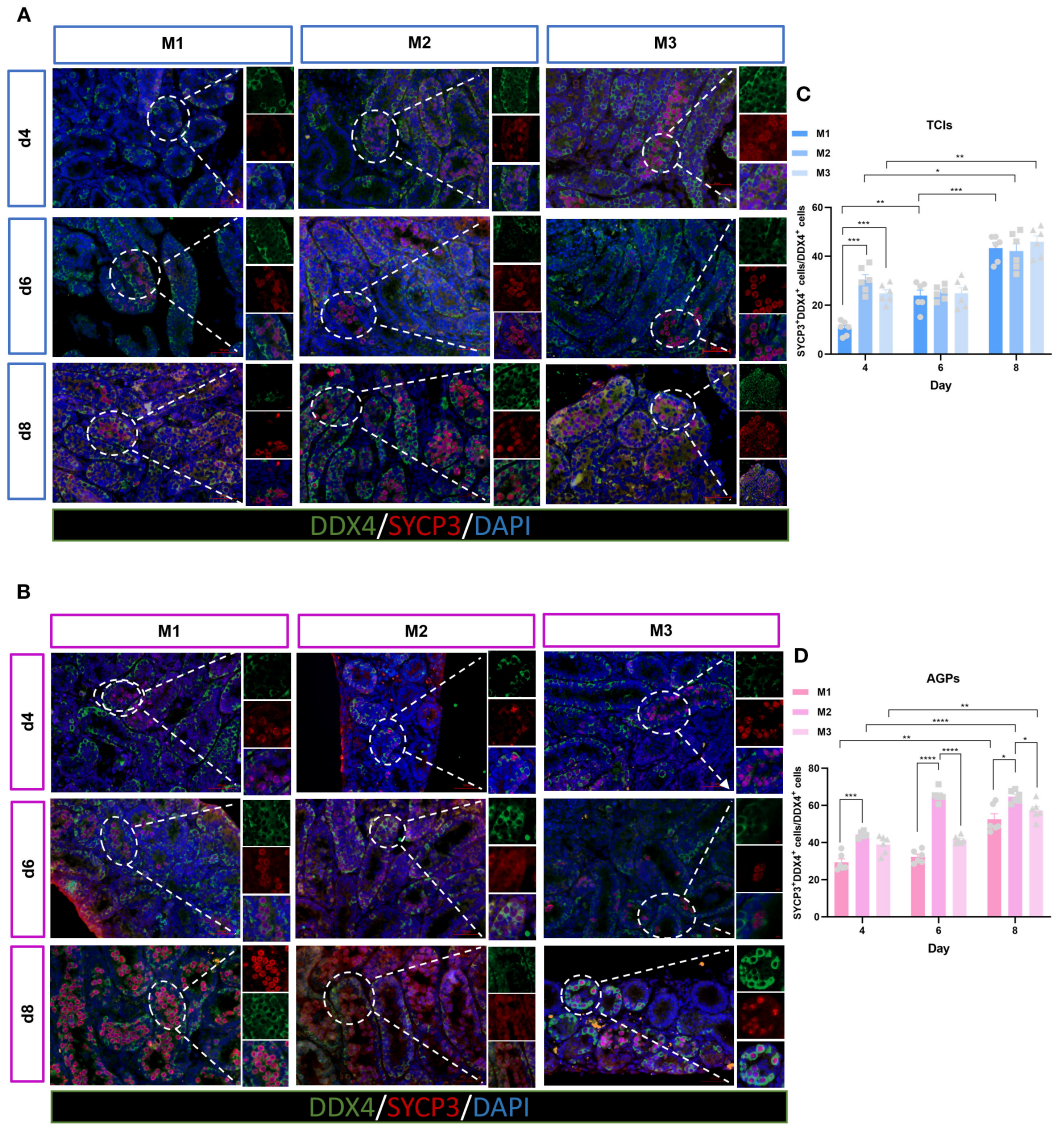
Evolution of germ cell differentiation over the culture period. **(A, B)** Tissue fragments were cultured in Tissue Culture Plant Insert **(A)**, and in AGPs **(B)**, immunofluorescence duplex staining for SYCP3^+^/DDX4^+^. **(C, D)** Evolution of the number of meiotic cells (SYCP3^+^), Percentage of SYCP3^+^ primary spermatocytes per total number of DDX4^+^ germ cells over the culture period. Images are shown at ×400 magnification. Scale bars = 50μm. Statistical significance was determined using ANOVA (*p ≤ 0.05, **p ≤ 0.01, ****p ≤ 0.0001).

We utilized SYCP3 as a marker for spermatocytes. SYCP3 is a meiosis-specific protein that constitutes an essential component of the synaptonemal complex, specifically labeling its axial/lateral elements during prophase I of the first meiotic division (27). In the **Figure 8**, the stage of testicular tissue development corresponding to the d4 M3 culture system during *in vitro* spermatogenesis is equivalent to the level of dpp9 day *in vivo*, at which time the SYCP3 positive signal is already present. During spermatogenesis *in vivo*, the positive signal of SYCP3 in the testis tissue of dpp10 Suckling mice was also observed in **Supplementary Figure 1**, which was consistent with the *in vivo* developmental process. This suggests that during the early stages of spermatogenesis, the progression of the first meiotic division remains largely consistent in both *in vivo* and *in vitro* systems. To investigate the differentiation and development of germ cells in *in vitro* cultured ITTs, we examined the presence of spermatocytes (SYCP3^+^) within the STs (**Figures 8A, B**). The results showed that both the culture system and duration had significant effects on the entry of germ cells into the meiotic phase. By day 4 of culture, spermatocytes were observed within the tubules. Interestingly, the M2 exhibited a significantly higher number of meiotic cells compared to the M1 and M3, with statistical significance. On days 6 and 8, the number of spermatocytes increased markedly across all three systems (**Figures 8C, D**). Our findings indicate that ITTs in all culture systems can progress into the meiotic phase during *in vitro* culture, with the M2 showing the most pronounced effect. This may also contribute to the above results of relatively lower quantity of spermatogonia while high proliferation of spermatogonia in M2. The differentiation into spermatocytes may limit their capacity for self-accumulation. In contrast, although M1 spermatogonia exhibited a certain degree of proliferative ability, their numbers remained relatively high. This may be due to their reduced differentiation and a greater focus on self-renewal or the maintenance of the spermatogonial pool.

## Discussion

Organotypic culture of testicular tissue serves as a significant *in vitro* model for different purposes (28). The gas-liquid interphase method is a classic approach widely used in testicular tissue culture (29). However, the optimal culture media and tools have yet to be determined, and their selection is often arbitrary. This study compared three basic culture media and two types of tools for their effects on ITT. Over an 8-day culture period, HE staining revealed apparently preserved morphology and tubular structures in all systems, with increasing tubular diameters indicating dynamic ST development. Spermatogonia were well-preserved, and germ cell numbers significantly increased. M2 and M3 showed higher germ cell proliferation than M1, with M2 having the highest activity but lower spermatogonia numbers. M1 had lower proliferation but better maintained spermatogonia stability. M2 promoted more spermatocyte differentiation, while M3 showed moderate performance with balanced proliferation and preservation. This study highlights the specific impacts and applications of different culture for ITTs.

Spermatogonial stem cells (SSCs), a subpopulation of spermatogonia located on the basement membrane of STs, are responsible for balancing self-renewal and differentiation to eventually produce haploid spermatozoa. SSCs comprise approximately 1.25% of the total spermatogonia population in mice (30, 31). Mouse SSCs are categorized into A single (As), A paired (Apr), and A aligned (Aal), collectively defined as undifferentiated spermatogonia (31). Zinc finger and BTB domain-containing protein 16 (ZBTB16), also known as PLZF, is predominantly expressed in undifferentiated spermatogonia, including As, Apr, and Aal4–16 subtypes (32). The presence of round spermatids was observed by Sato et al. in FBS-supplemented ITT, although no elongated sperm were identified (33). In our culture system, we observed that spermatogonia were better maintained in the FBS-containing M1 culture system, demonstrating the improved maintenance of SSCs in the M1. The maintenance and development of germ cells (DDX4^+^) are crucial for fertility; we found that the number of germ cells was well-maintained across all three culture systems as the culture duration increased. And further analysis of germ cell proliferation revealed that proliferation activity was more pronounced in the M2 and M3.

In mouse testes, undifferentiated spermatogonia possess stem cell potential, playing a critical role in self-renewal and proliferation to generate more spermatogonia. This population of cells is fundamental to spermatogenesis (34). In this study, we assessed the proliferation efficiency of spermatogonia using dual immunofluorescence staining for SALL4^+^+PCNA^+^/SALL4^+^ and PLZF^+^+KI67^+^/PLZF^+^. The transcription factor Sal-like 4 (SALL4), a member of the zinc finger Sal gene family, is associated with stem cell maintenance and organ patterning during embryonic development. Postnatally, its expression is restricted to the gonads, particularly in undifferentiated spermatogonia of adult testes (26). During *in vitro* culture, regardless of the tools used, spermatogonia in M2 and M3 exhibited relatively active proliferation. Notably, numerous proliferating (KI67^+^) cells were observed within the tubules in M2. In contrast, spermatogonial proliferation was less active in M1, with lower germ cell proliferation rates. The enhanced proliferation observed in M2 and M3 was attributed to the presence of KSR in the culture medium. KSR contains defined components, such as growth factors and hormones, and is widely used to culture various stem cells, supporting their proliferation *in vitro* (35). However, the specific composition of KSR has not been fully disclosed, leaving the roles of individual components unclear. The proliferation rate of spermatogonia was higher in M2, but the actual number of spermatogonia did not increase, which may be due to compensatory proliferation, and the remaining spermatogonial stem cells in the lumen accelerated proliferation to compensate for the loss (36, 37).

Studies have shown that early (pre-)leptotene spermatocytes can appear in the STs of 10dpp mice (38). In this study, we used testicular tissue from 5dpp mice, and therefore began observing ITT from day 4 of culture to determine whether meiosis had been initiated. The results revealed that tissues from all three culture systems entered meiosis by day 4. Over time, a large number of spermatocytes were observed within the tubules, indicating that ITT cultured *in vitro* can progress into the meiotic phase. In three systems, M2 showed more spermatocytes, suggesting that M2 may promote tissue differentiation, making it more favorable for entry into the meiotic phase.

Sertoli cells provide a supportive microenvironment essential for the development and maturation of germ cells during spermatogenesis (39). GATA4, an important transcription factor, is widely used as a marker for Sertoli cells and is detectable at all stages of Sertoli cell development (40). During *in vitro* culture, we observed a decrease in the mm² tubule/GATA4^+^ ratio with prolonged culture time. However, the actual number of Sertoli cells was well-maintained throughout the culture period. We analyzed the increasing diameter of STs and the growing number of germ cells within the tubules over time. These factors led to a decreased proportion of Sertoli cells within a fixed area, rather than a true reduction in the number of Sertoli cells. Further supporting our findings, our data (**Supplementary Figure 2**) on the growth and development of Sertoli cells in mice confirm this perspective. Prepubertal Sertoli cells in mice remain proliferative, and by dpp14 days post-partum, Sertoli cells reach maturity and cease proliferation (41).

**Figure 9:**
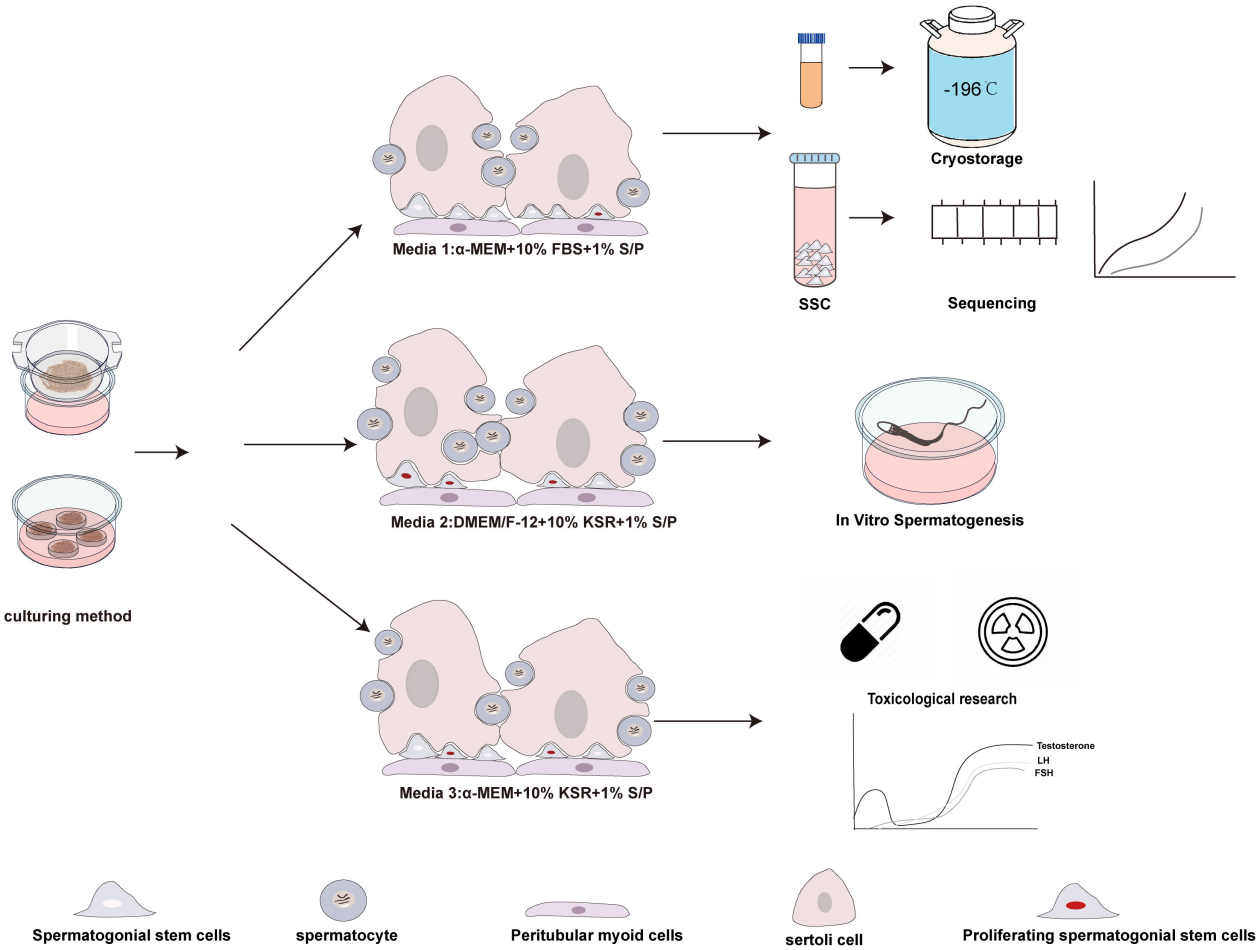
Potential applications of the three cultivation systems based on their characteristics.

Our results demonstrated that different culture systems exhibit distinct characteristics in terms of spermatogonia proliferation, differentiation, and maintenance, making them suitable for various research purposes. The M1 system better maintained spermatogonia numbers, with lower proliferation rates for both germ cells and spermatogonia. Overall, the M1 system showed more stable characteristics, making it a promising choice for cryopreservation and storage of immature testicular tissue, preserving SSCs that serve as the foundation for *in vitro* spermatogenesis. Additionally, M1 may facilitate the enrichment of spermatogonia, providing a basis for further exploration of spermatogonial fate in organotypic culture using sequencing technologies. The M2 medium demonstrated an increased population of spermatocytes (SYCP3^+^), suggesting its superior potential as a basal medium for *in vitro* spermatogenesis. Further supplementation with pro-differentiation factors to optimize this culture system may significantly advance research in *in vitro* sperm production. The M3 retained a certain number of spermatogonia while also supporting germ cell and spermatogonial proliferation. This dual characteristic makes M3 a better choice for applications like drug screening and toxicological studies. For example, chemotherapy drugs targeting specific phases of the cell cycle, which are particularly effective against proliferating cells, can be studied using M3 to assess their impact on proliferative spermatogonia. Additionally, M3 is suitable for studying testosterone secretion and testicular endocrine function, including screening for drugs that influence steroid hormone synthesis. Overall, our findings provide tailored recommendations for the use of different culture systems in organotypic culture of testicular tissue, offering more suitable platforms for various research objectives (**Figure 9**), and enhance the reliability of experimental outcomes. Although we have conducted a solid exploration of organotypic culture methods for prepubertal mice and provided a comprehensive histological characterization, we did not conduct subcellular-level analyses or systematically assess the progression of meiosis and the specific culture conditions that most effectively support spermatocyte development during the first meiotic division. These limitations constrain our ability to draw definitive conclusions about the capacity of these culture systems to support complete spermatogenesis, which involves two consecutive meiotic divisions.

In summary, this study demonstrated that testicular tissue exhibits distinct developmental characteristics across different culture systems. Specifically, spermatogonia in the M2 showed remarkable proliferation and differentiation potential, while those in the M1 excelled in stability and self-renewal. The differences in testicular tissue between the two culture tools were minimal. These findings highlight the importance of selecting an appropriate culture system based on specific research objectives, underscoring its significance in studies utilizing organotypic culture of testicular tissue.

This study established a robust foundation for future advancements in reproductive research and the development of treatment methodologies. These findings provide a customizable platform for fertility preservation and offer novel strategies for spermatogenic disorders. This platform not only enables precise toxicological screening but also serves as a research model for testicular development, facilitating the exploration of factors influencing *in vitro* spermatogenesis. Furthermore, it provides a research framework for regenerative medicine, such as the optimization of culture media for *in vitro* spermatogenesis, thereby holding significant implications for understanding and treating male infertility.

## Data Availability

The original contributions presented in the study are included in the article/**Supplementary Material**. Further inquiries can be directed to the corresponding authors.

## Supplementary material

Supplementary Figure 1 Negative and positive controls in all experimental groups.

Supplementary Figure 2 Growth and development of mouse Sertoli cells. **(A, C)** Immunofluorescence staining of GATA4/SOX9 Positive Cells. **(B, D)** Evolution of the number of Sertoli cells mm²in the ST of mice at 3dpp,5dpp,8dpp,10dpp. Images are shown at ×400 magnification. Scale bars = 50μm. Statistical significance was determined using ANOVA (*p ≤ 0.05, **p ≤ 0.01, ****p ≤ 0.0001).

Supplementary Figure 3 Evolution of spermatogonia cell proliferation over the culture period. **(A, B)** Tissue fragments were cultured in Tissue Culture Plant Insert **(A)**, and in AGPs **(B)**, immunofluorescence duplex staining for SALL4^+^/PCNA^+^. **(C, D)** Evolution of the number of proliferating Spermatogonia cells (SALL4^+^+PCNA^+^/PCNA^+^) over the culture period. Images are shown at ×400 magnification. Scale bars = 50μm. Statistical significance was determined using ANOVA (*p ≤ 0.05, **p ≤ 0.01, ****p ≤ 0.0001).
