# Supplementary figures and images for: Organotypic culture system for prepubertal mice testicular tissue: A comparative study
Source: Front Endocrinol (Lausanne). 2025 Sep 25;16:1664628. doi: 10.3389/fendo.2025.1664628 (PMC12507631; doi:10.3389/fendo.2025.1664628)

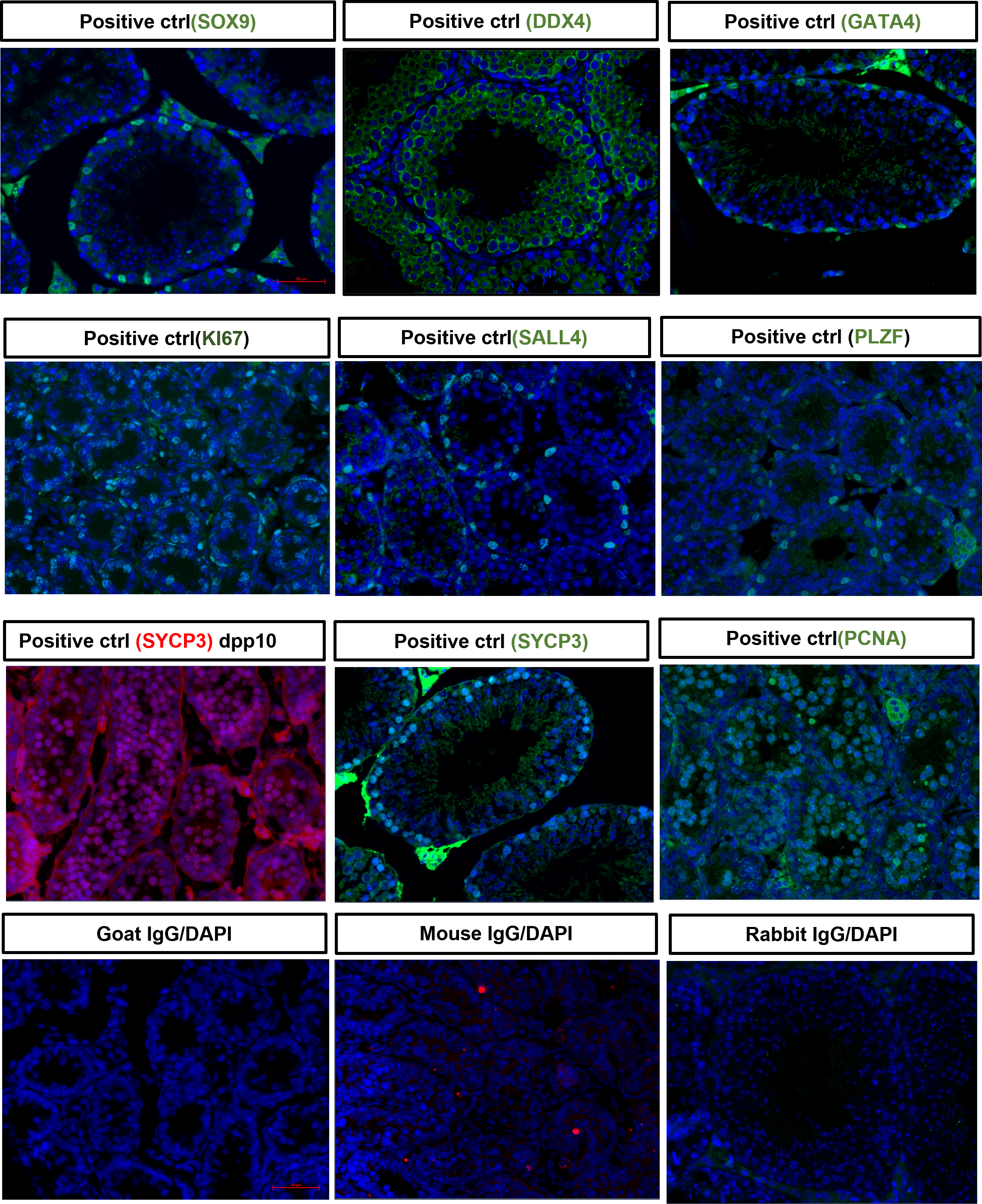

Supplement: Supplementary Figure 1 — Negative and positive controls in all experimental groups. [file Image1.tif]

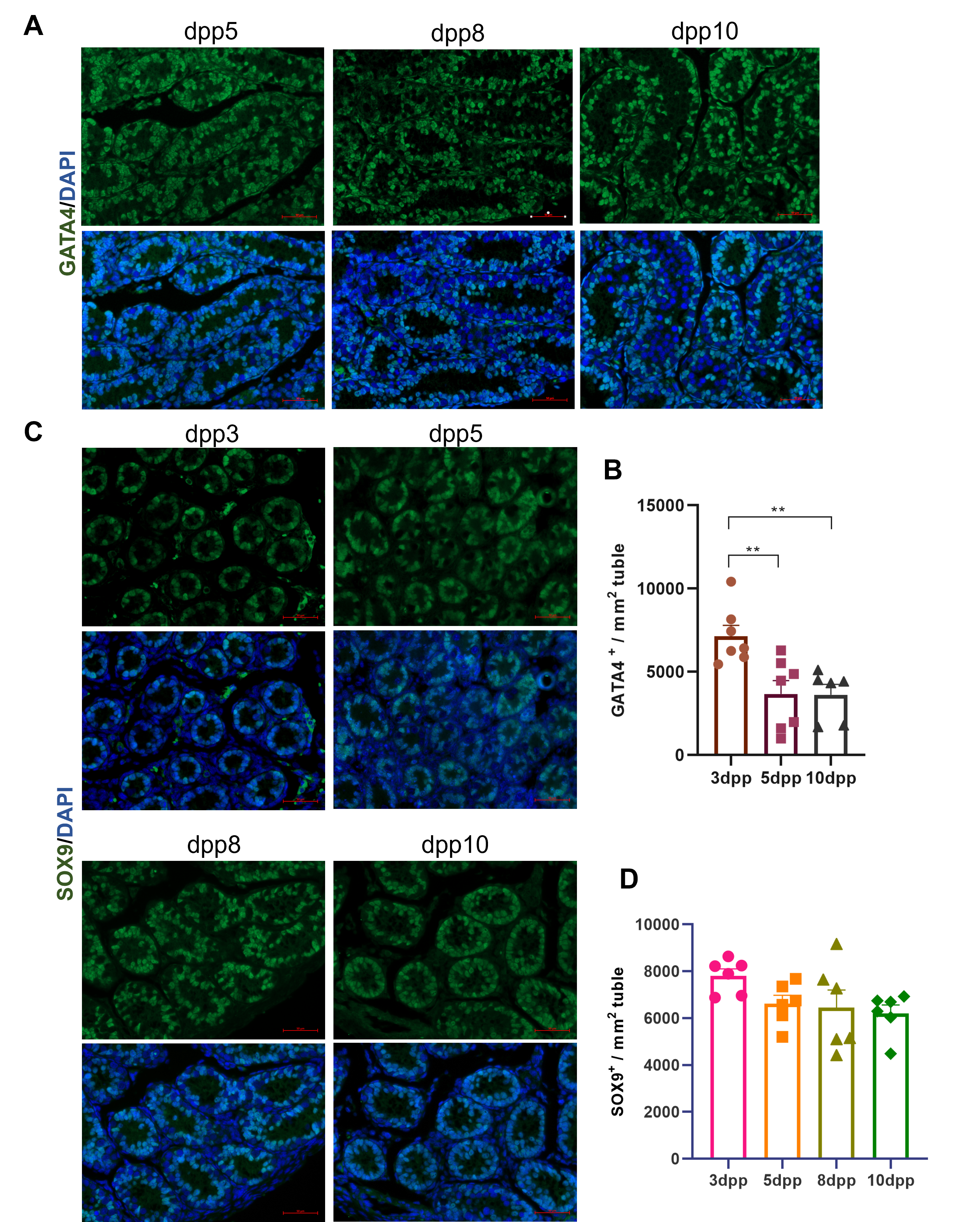

Supplement: Supplementary Figure 2 — Growth and development of mouse Sertoli cells. (A, C) Immunofluorescence staining of GATA4/SOX9 Positive Cells. (B, D) Evolution of the number of Sertoli cells mm²in the ST of mice at 3dpp,5dpp,8dpp,10dpp. Images are shown at ×400 magnification. Scale bars = 50μm. Statistical significance was determined using ANOVA (*p ≤ 0.05, **p ≤ 0.01, ****p ≤ 0.0001). [file Image2.tif]

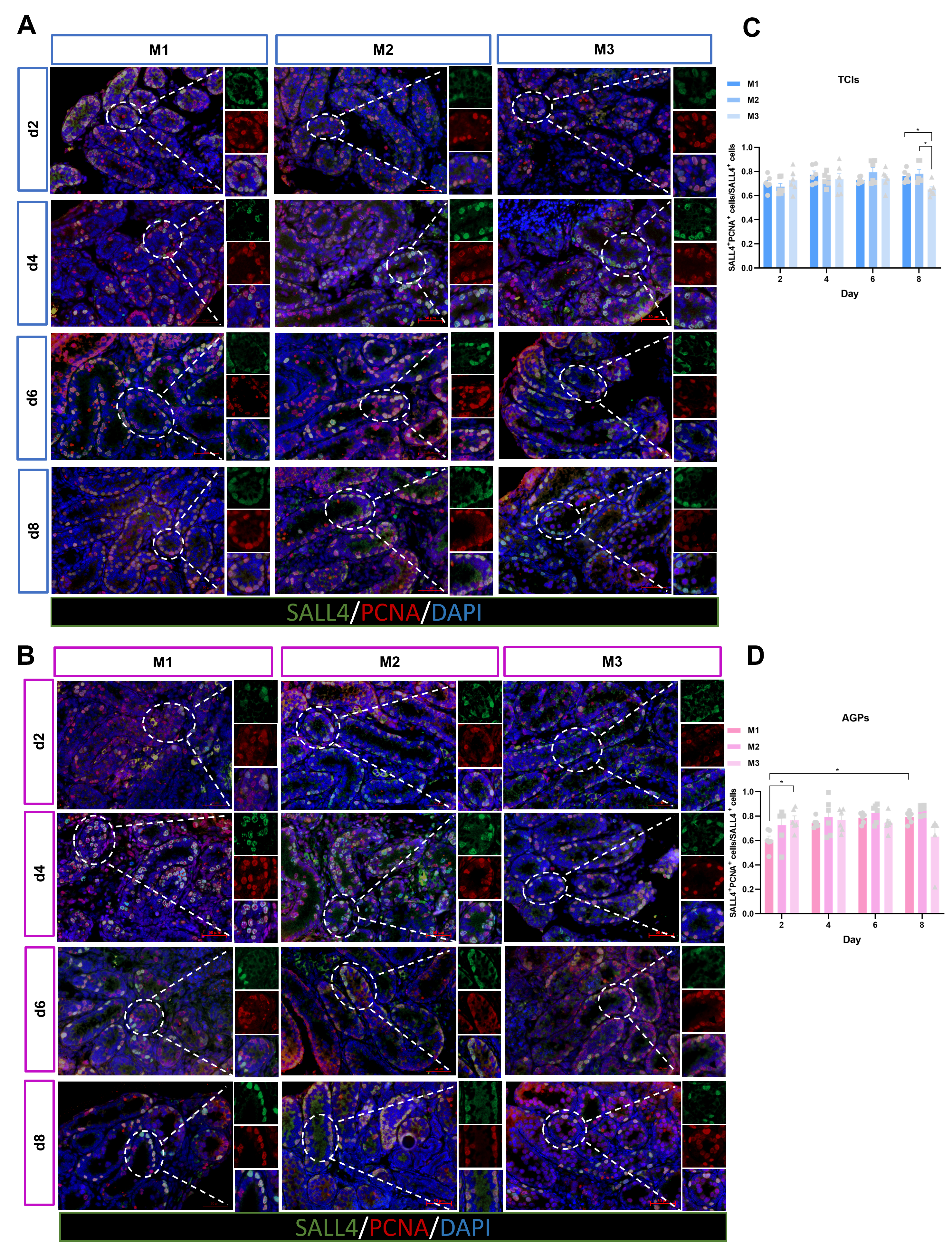

Supplement: Supplementary Figure 3 — Evolution of spermatogonia cell proliferation over the culture period. (A, B) Tissue fragments were cultured in Tissue Culture Plant Insert (A), and in AGPs (B), immunofluorescence duplex staining for SALL4+/PCNA+. (C, D) Evolution of the number of proliferating Spermatogonia cells (SALL4++PCNA+/PCNA+) over the culture period. Images are shown at ×400 magnification. Scale bars = 50μm. Statistical significance was determined using ANOVA (*p ≤ 0.05, **p ≤ 0.01, ****p ≤ 0.0001). [file Image3.tif]
