# Supplementary material for: Organotypic culture system for prepubertal mice testicular tissue: A comparative study
Source: Front Endocrinol (Lausanne). 2025 Sep 25;16:1664628. doi: 10.3389/fendo.2025.1664628 (PMC12507631; doi:10.3389/fendo.2025.1664628)
Supplement: Supplementary file 4 [file Table1.docx]

**Supplementary table1**:

| **Protein** | **Target** | **Primary antibody** | **Primary antibody type** | **Primary antibody dilution** |
| --- | --- | --- | --- | --- |
| **DDX4** | **Germ cells** | **ABclonal-A15624** | **Rabbit pAb** | **1:100** |
| **DDX4** | **Germ cells** | **Abcam-AB180462** | **Mouse mAb** | **1:400** |
| **GATA4** | **Sertoli cells** | **ABclonal-A4306** | **Rabbit pAb** | **1:100** |
| **SALL4** | **Spermatogonia** | **ABclonal-A16193** | **Rabbit pAb** | **1:100** |
| **PLZF** | **Spermatogonia** | **R＆D-AF2944** | **Goat pAb** | **1:200** |
| **SYCP3** | **Meiotic cells** | **Santa-sc74569** | **Mouse mAb** | **1:100** |
| **KI67** | **Proliferating cells** | **C.S.T-9129T** | **Rabbit mAb** | **1:400** |
| **PCNA** | **Proliferating germ cells** | **Santa-SC56** | **Mouse mAb** | **1:100** |
